# The Sum of Fears in Cancer Patients Inside the Context of the COVID-19

**DOI:** 10.3389/fpsyt.2021.557834

**Published:** 2021-04-07

**Authors:** Lucas Bandinelli, Felipe Ornell, Lisia von Diemen, Felix Henrique Paim Kessler

**Affiliations:** ^1^Postgraduate Program in Psychology, Pontifícia Universidade Católica do Rio Grande do Sul, Porto Alegre, Brazil; ^2^Developmental Cognitive Neuroscience Lab (DCNL), Pontifícia Universidade Católica do Rio Grande do Sul, Porto Alegre, Brazil; ^3^Center for Drug and Alcohol Research and Collaborating Center on Alcohol and Drugs, Hospital de Clínicas de Porto Alegre, Universidade Federal do Rio Grande do Sul, Porto Alegre, Brazil; ^4^Graduate Program in Psychiatry and Behavioral Sciences, Universidade Federal do Rio Grande do Sul, Porto Alegre, Brazil; ^5^Queen's University Department of Psychiatry, Providence Care Hospital, Kingston, ON, Canada

**Keywords:** cancer, fear, mental health, COVID-19, pandemic (COVID-19)

## Abstract

The pandemic resulting from COVID-19 has led to the collapse of the health system in dozens of countries. Parallel to clinical risk, the appearance or intensification of psychiatric symptoms has also been documented. The identification of groups at risk is essential for the establishment of preventive and therapeutic strategies. Cancer patients appear to be especially vulnerable both from a clinical and psychiatric perspective. Problems related to contamination and the cancer treatments themselves are intertwined, causing a sum of patients' fears to arise, which can cause mental effects. This study aims to review and investigate the impact of COVID-19 on the mental health of cancer patients and indicate possible support strategies.

## Introduction

The 2019 coronavirus (COVID-19) pandemic is an international public health emergency unprecedented in the 21st century (1). Despite the fact that most of the contaminated are asymptomatic, the disease can have a serious evolution, with the occurrence of respiratory failure and the need for support in intensive care units (ICU) (2). The severity of the disease is very heterogenous, and elderly people suffering from chronic diseases and those with immunosuppression are at the highest risk (3). In particular, cancer patients may be a vulnerable group to morbimortality by this infectious disease (4) and to higher levels of stress than the general population.

Infectious diseases can be a vital threat to cancer patients, so this relationship has been extensively investigated in recent decades (5, 6). During the H1N1 pandemic in 2009, for example, a study found a high incidence of hospitalization, severe pneumonia, admission to the ICU, mechanical ventilation, and mortality in cancer patients (7). Currently, during the COVID-19 pandemic, there is preliminary evidence that cancer patients may be particularly susceptible to contamination (8–12). In addition, recent systematic reviews have shown that cancer may be associated with the worsening of the disease (4, 13) and an increased risk of death (14, 15). This sum of factors can generate additional stress and intense suffering in a population that already has several mental health issues (16).

It is well-described that in cancer patients, the rates of problems related to mental health are higher than that evidenced in the general population (17). The implications of this can affect even the clinical prognosis. A recent meta-analysis reported that symptoms of depression and anxiety can affect prognosis, being related to reduced survival and increased mortality (18).

During the pandemic, cancer patients may be experiencing an intensification of psychological distress (19). There is evidence showing that the rate of cancer patients in need of mental health attention has increased in this period (20). Despite this, it is observed that access to psychological and psychiatric treatment in this population can be impaired during the pandemic (16, 19). The World Health Organization (WHO) estimates that despite the growing demand, the pandemic has disrupted mental health services in 93% of countries worldwide (1). On the one hand, it is observed that the reorganization of the health system to meet the demand of patients with COVID-19 may have compromised access to cancer services and drugs (21). On the other hand, fear of virus contamination may lead to avoidance of hospital environments (22–24). In both cases, this can cause a delay in treatment and worsen the prognosis. In addition to clinical issues, this may be associated with high levels of distress and mental suffering (22, 23, 25, 26). In this sense, risks and fears add up, generating a question: What is the impact of the sum of these fears on the mental health of cancer patients? To better understand this phenomenon and raise new insights on the subject, we conducted a short narrative review on the topic.

## The Mental Burden in Cancer Patients

Problems related to mental health in patients with cancer and greater psychological vulnerability are well-described in the literature (27). Going through a major life-stressing event, as in the case of discovering an oncological diagnosis, brings a series of emotional reactions increasing the perceived stress load (28). This increased burden often makes patients experience difficulties in returning to an emotional state prior to the discovery of the disease, impacting their quality of life (29).

Some mental disorders are more recurrent in the cancer population, as in the case of depression and anxiety, where their rates are higher than those observed in the general population (27, 28, 30–33). It is estimated that around 58% of cancer patients have some form of depression during treatment, ranging from mild depressive symptoms to the clinical diagnosis of major depressive disorder (34). To assess how common depressive symptoms may be during cancer treatment, a systematic review followed by a meta-analysis revealed that the presence of a major depressive disorder was described in 15% of studies and minor depression in 20% (31). The prevalence rates of depression in the oncology population may show some inconsistency and vary according to the method used for its assessment (35).

Concerning anxiety disorders, it is estimated that 19% of patients have clinical symptoms of anxiety, while 23% have subclinical symptoms once they do not fulfill all diagnostic criteria for some specific types of anxiety disorder (32). One of the phases during the continuum of cancer treatment that most arouses anxiety symptoms is the diagnosis (36). It is in the initial moments when the patient still needs to assimilate the information and adapt to different conditions that involve his treatment and make countless decisions that his anxiety is exacerbated (37, 38). Even after the treatment period, the symptoms of anxiety and depression can still be present for up to 10 years after the diagnosis of the disease (39).

Some factors contribute to the more significant presence of emotional problems in oncology patients, like the type of cancer (32). The highest prevalence of emotional distress appears to occur in patients with breast cancer, followed by patients with head and neck cancer (40, 41). Also noteworthy is the presence of depressive symptoms in patients with lung cancer, where the presence of guilt due to risky behaviors—such as constant use of tobacco—is shown to be an influential factor in altering mood (42, 43).

The stage of the disease during the diagnosis period was also one of the factors that influences the change in the patients' emotional state. People who discovered the disease at a very advanced stage have higher rates of distress (44), mainly because they have higher pain rates (45). The type of treatment performed also influences the prevalence rates of emotional problems. Treatments that require more invasive procedures, as in the case of surgeries, can contribute to the burden of distress and worsen the quality of life (46). The use of some chemotherapeutic drugs can have a deleterious effect on the cognitive functions of patients causing them to have problems involving memory and concentration (47, 48). In addition, the presence of side effects such as nausea and fatigue can lead to an increase in anxiety and depressive symptoms (49, 50).

After the treatment ends, the impact of cancer on the mental health of patients may persist, mainly due to the presence of fear that the disease may return. A previous study, for example, showed that the stress resulting from fear of death and recurrence affects between 22 and 87% of cancer patients, which can lead to neurobiological, emotional (51), and behavioral changes (52). Fear of disease recurrence can keep patients in a constant state of alert, mainly due to cognitive biases for stimuli considered threatening (for example, some pain in the body), impacting their quality of life (53). Thus, cancer patients are particularly vulnerable to emotional problems, in the face of the pandemic, and all these symptoms can be intensified.

## The Impact of COVID-19 in Cancer Patients

Patients undergoing cancer treatment, especially in cases where the use of chemotherapy and/or immunotherapy is necessary, may be especially vulnerable to an increased risk of infection during the pandemic (54). Multiple risk factors, such as the existence of clinical comorbidities and poor functional status are frequently seen in patients with cancer. In addition, there is impairment of immunity due to the malignancy of the disease or to antineoplastic therapy (5). It is also noteworthy that different types of cancer produce immunological suppression in different extensions, as in the case of onco-hematological diseases (25). These risk factors often lead to frequent visits to hospitals to treat the disease or other concomitant medical conditions (or those resulting from the condition), which may increase the risk of contamination (5, 55). However, it is observed that the fear of exposure to the virus when attending hospital environments can also lead patients to interrupt treatment or neglect symptoms (22–24, 56). Still, it is necessary to consider that this group may be affected by the scarcity of essential medicines, and may suffer from the reduction of health activities, in the community, due to the implementation of the social distancing and lockdown guidelines (6, 9). In some cases, patients may encounter difficulties in carrying out important procedures for their treatments, such as in the case of surgery, due to the high demand for hospitalization due to the pandemic (57), which may further increase the fear of the progression of the oncological disease (26, 58). In addition, a systematic review recently demonstrated that during a pandemic, delay in surgery can reduce survival (59).

In this sense, fear of contamination, or the effects of the pandemic on health and treatment, can lead to additional potential stressors in the oncology population. Thus, in addition to the biological risk and the reduction in treatment offerings, it is necessary to take the influence of COVID-19 on the mental health of this population seriously. Moreover, this factor can be enhanced by the removal of family members and social support, which may be deficient, especially in the lower classes (6, 60, 61), who may also have difficulty accessing remote consultations (62). In the case of hospitalization, it is common for patients to be alone to reduce the risk of contagion, which can cause anxiety, sadness, feelings of abandonment, and the fear of dying alone (63). These manifestations can also appear in quarantined patients, whose psychological distress due to loneliness can be aggravated (64). Thus, it is observed that several factors influence the emotional aspects of patients during this period, resulting in the sum of the fear of different issues, further damaging the mental health of this population.

## Mental Burden in Cancer Patients: the Sum of Fears

During the pandemic, the fear of contamination, the difficulty of accessing treatment, and distance from family have added to the fear of death or a clinical condition worsening, which can intensify the feeling of stress even more. In risk groups, such as patients with serious or terminal illnesses, these symptoms interact with those of the current illness and can be even more intense. Depressive, anxiety (including panic attacks and posttraumatic stress), psychotic and paranoid disorders, or even suicidal behaviors can emerge (65, 66).

It should be noted that recent publications consider that, simultaneous with the COVID-19 pandemic, there seems to be a “fear pandemic” affecting the general population, becoming a trigger for the worsening of anxiety symptoms (67, 68). The fear of being contaminated and the drastic changes in daily routines can already be considered important stressors, but infected patients (or those with suspected infection) can manifest intense emotional and behavioral reactions (64, 69). One study found that the rates of fear and anxiety in cancer patients during the pandemic are high, and according to the literature, breast and lung cancer patients had the highest rates (70).

Undergoing cancer treatment alone generates numerous fears in patients, mainly involving fear of death (71). As mentioned earlier, during the COVID-19 pandemic, many patients may experience difficulties in accessing their treatments, causing the fear of cancer to progress even more (26, 58). In this sense, fear of being infected by the virus is added to the fear that their underlying diseases may become worse due to the lack of adequate treatment (72). The sum of these fears can cause a constant sensation of alertness (53) and that in cancer patients, this is potentially harmful, since stress hormones can activate oncogenic viruses and alter various aspects of the immune function (73). In addition, uncertainty about infection and death or about infecting family and friends can induce dysphoric mental states, including irritability and aggression under a sense of being experiencing something terrifying (66, 69, 74).

**Figure 1:**
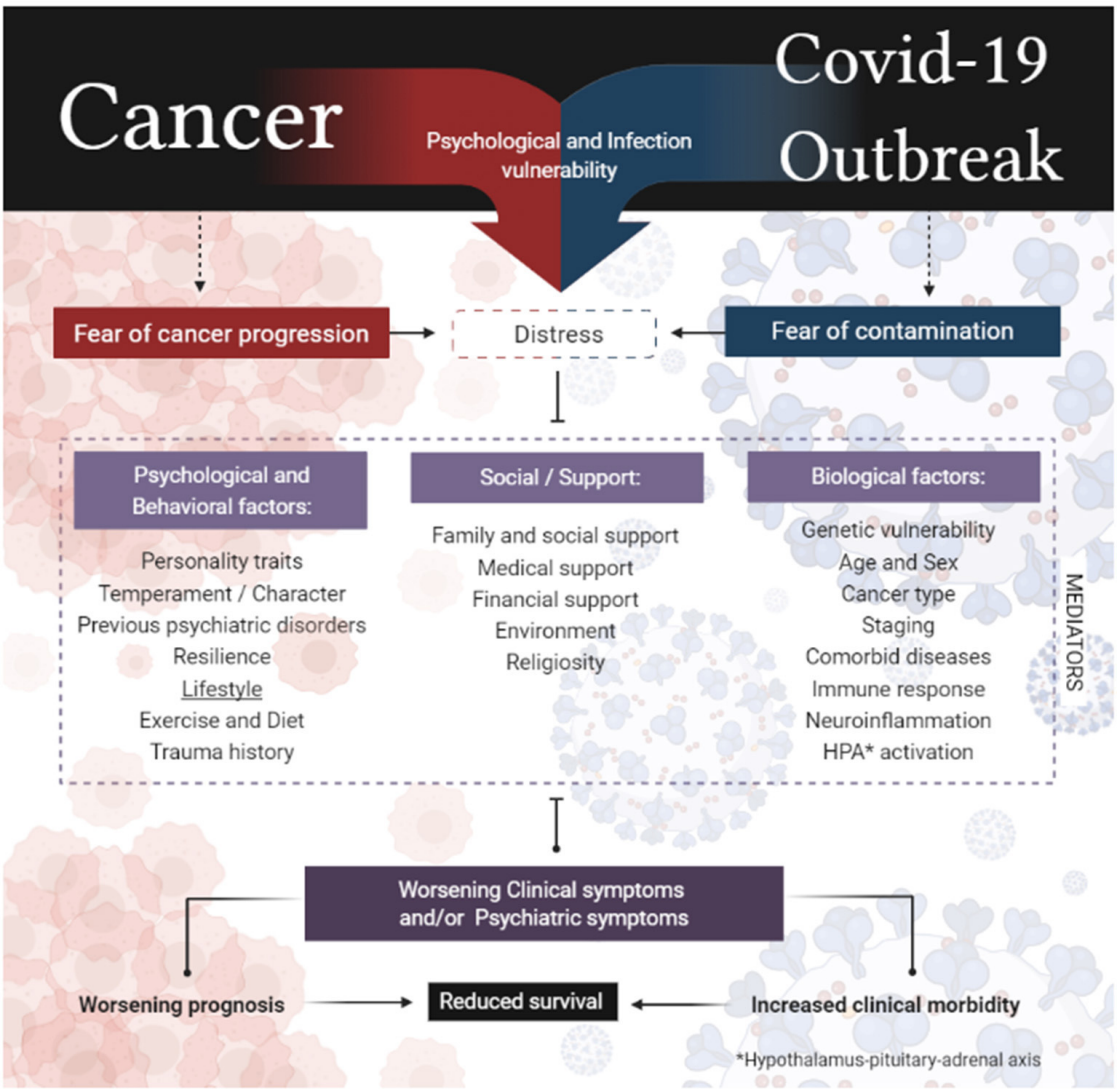
Interactive model between the sum of fears in cancer patients.

Fear is a natural reaction to threatening stimuli and triggers a series of biopsychological responses that prepare the individual for fight or flight reactions (75). This physiological response to stressful events involves the central nervous system (CNS) by activating the autonomic nervous system (ANS) and the hypothalamus–pituitary–adrenal axis (HPA) (76). In addition to preexisting vulnerability factors such as genetic, environmental, and gender differences, hyperactivation of the HPA axis can lead to an increase in cortisol, which can trigger a series of psychological problems, such as the onset of panic attacks and increased general anxiety levels (77–79). Some explanatory models for these outcomes such as the second wave hit model (80) and the bidirectional multi-system reactivity hypothesis (81) theorize that the interaction between a preexisting vulnerability with stressful events capable of triggering strong biopsychological reactions can lead to activation of complex systems and signaling pathways that can contribute to the onset of deleterious psychological symptoms, mainly due to responses to major stressful events, as in the case of COVID-19. In addition, the constant activation of HPA can result in the appearance of depressive symptoms, mainly due to responses to threatening events, as in the case of COVID-19 (74, 82, 83). It is clearly not possible to affirm the direct connection between the events, and neither would this be our proposal, but, nevertheless, it is necessary to reflect on the possibilities of the sum of factors leading to negative outcomes in this specific population. Thus, Figure 1 presents an interactive model between the events, clarifying the possible psychological response to the sum of fears.

## Perspectives of Mental Health Care of Cancer Patients During the Pandemic

Sadness and depressive symptoms can be recurrent and are associated with negative and catastrophic thoughts in cancer patients during the pandemic. They may occur with discouragement or despair that is related to a possible relapse or infection with the new coronavirus. This can make cancer patients even more vulnerable, with a greater tendency to develop physical problems (for example, impaired immunity) or emotional problems (for example, high levels of anxiety and depression) (24, 58, 84).

Thus, it is essential that the institutions responsible for cancer treatments set up multidisciplinary crisis committees to develop and update the guidelines and strategies of mental health care for this population during the pandemic. In a recent study it was shown that despite the fact that we are aware that the crisis is having a significant impact on the mental health of cancer patients, few are being monitored or receiving specific mental health care (19). It is also essential that telephone or Internet service channels are urgently implemented (9).

Telemedicine is not exactly a novelty in oncology, and it has shown satisfactory results, especially for patients living in remote areas (85). In the context of mental health, the consolidation of remote care by professionals in the field during the pandemic has been observed (86). For patients in isolation, telephone and Internet calls are crucial alternatives to guarantee access to treatment and reduce the risk of COVID-19 transmission (87).

The application of screening protocols by telephone is a valid strategy for identifying cancer patients that are in severe mental distress. This can help to track patients at risk and direct support measures that reduce the intensity of symptoms and suffering (20). Recently, a panel of psychiatrists from 15 countries developed a protocol for providing mental health care during the pandemic. The protocol provides for an initial semistructured assessment and a series of interventions according to the degree of symptom intensity. All interventions follow evidence-based adequacy and efficacy criteria and may vary from psychoeducation to emergency care. This protocol can serve as a starting point for the development of strategies according to the region and the public served (88). Within this perspective, strategies need to contemplate four aspects: (1) dissemination of information (which involves communication and psychoeducational content), (2) counseling, (3) emergency support (psychological first aid), and (4) structured and more longitudinal interventions (68). However, as previously mentioned, not all patients have the possibility of having access to remote services, requiring care alternatives for this population to be considered (62). Thus, we take the opportunity to describe in Table 1 some strategies that cancer patients can use to manage the emotional effects resulting from the pandemic, thinking about their self-care.

**Table 1:** Recommendations for self-care in mental health for cancer patients.

**Exhibited problem**	What to do^*^
Anxiety	Use breathing techniques as the diaphragmatic breathing or any other of your preference to control some physical symptoms of anxiety (tachycardia, psychomotor agitation, shortness of breath, and accelerated breathing) (89).
	Try to separate the problems in two categories: the ones you can control and the ones that you cannot control. Focus on those problems which you have control over and search for strategies of problem resolution for them (90).
	Try to distract from news regarding COVID-19, restricting access to information (91).
Depressive mood	Try to listen to pleasant songs of your preference, which increase the feeling of pleasure and well-being (92).
	Do physical activities within your possibilities and reality of oncologic treatment: light walks, Yoga practice, and meditation (93).
	Keep in touch with friends and relatives even if it is a long distance. Remember that by talking to them, you do not necessarily need to speak about your treatment or the pandemic. Choose subjects that are convenient and bring you a feeling of joy (94).
Excessive Fear	Think about how experiences related to this feeling have brought you consequences, and try to identify triggers that bring you this sensation (95).
	Practice mindfulness techniques to focus on the here and now, without letting your thoughts regarding the future get in the way of the present moment (96).
	Determine which are your beliefs and thoughts related to fear, as for example: “I believe that my future is going to be horrible”; “I am not going to endure what will happen”; “I am certainly going to get contaminated by the coronavirus.” After determining them, search and discuss with other people logical answers to those feelings, taking into account the probability of them actually happening and measuring how many of them are truly based on facts and not only on sensations (97).
	Remember all the situations in which you felt fear and were able to overcome it, analyzing what was the outcome of that situation and the emotional consequences you had.
	Make a chart with two columns: on the first write down all your worries, and in the second one write possible solutions for each (97).
	Talk to friends and close people in order to think together of all possible solutions for the problems you listed. In case you cannot find solutions, think about the impact that this problem is going to generate and about what resources you and other people may have to cope with it (97).
Sleep problems	Do sleep hygiene, searching for factors that may be contributing to this problem, such as heavy eating at night, excess intake of caffeinated drinks, room with too much luminosity and noise, etc. Determine the factor and search for solutions (98).
	If before going to sleep you keep thinking about your problems and cannot disconnect, try to postpone your thoughts. Make a deal with yourself and say you will only worry about your problems on the next day, when you wake up. Do not try to not think about your problems because this is going to make it worse, and bring your focus to the problem. Due to this reason, only say that you will postpone your worry (97).

* *For more information, see the references below. In case of greater severity, consider referral to a health professional*.

## Conclusion

In cancer patients, the rates of mental disorders are higher than those seen in the general population. Previously, it was observed that anxiety and depression are related to the clinical prognosis, even increasing the morbimortality rate. During the pandemic, there may be an aggravation of mental suffering, resulting from the sum of the fear of being contaminated by the new virus plus the fear of the progression of the oncological disease resulting from the gaps related to clinical care, as well as distance from loved ones. The effects of this sum of fears in cancer patients can interact with complex systems involving hyperactivation of the HPA system, among other things, increasing the sense of threat and affecting the quality of life of patients. Given this scenario, it is relevant to recognize the challenges of caring for cancer patients during the pandemic. Thus, it is essential that psychiatric evaluation and psychological support measures are implemented, which may also involve telemedicine, which can be useful for tracking patients at risk, identifying the degree of severity of symptoms, and implementing support strategies considering the social context.

## Author Contributions

LB and FO conceptualized, designed, and drafted the manuscript. LvD and FK provided critical revisions of the manuscript. All authors contributed to the article and approved the submitted version.

## Conflict of Interest

The authors declare that the research was conducted in the absence of any commercial or financial relationships that could be construed as a potential conflict of interest.
